# Quantitative benchmarking of iodine imaging for two CT spectral imaging technologies: a phantom study

**DOI:** 10.1186/s41747-021-00224-2

**Published:** 2021-06-23

**Authors:** Vanja Harsaker, Kristin Jensen, Hilde Kjernlie Andersen, Anne Catrine Martinsen

**Affiliations:** 1 Department of Life Sciences and Health, Oslo Metropolitan University, Box 4, St. Olavs plass, 0130 Oslo, Norway; 2 The Department of Diagnostic Physics, Oslo University Hospital, Bygg 20, Gaustad Sykehus, Box 4959 Nydalen, 0424 Oslo, Norway; 3 Sunnaas Rehabilitation Hospital, Bjornemyrvn. 11, 1453 Bjornemyr, Norway

**Keywords:** Contrast media, Iodine, Phantoms (imaging), Tomography (x-ray, computed)

## Abstract

**Background:**

The aim of this study was to quantitatively benchmark iodine imaging across specific virtual monoenergetic energy levels, iodine maps and virtual non-contrast images with different phantom sizes and iodine concentrations, using a rapid switching dual-energy CT (DECT) and a dual source DECT, in order to investigate accuracy and potential differences between the technologies.

**Methods:**

Solutions of iodine contrast (10, 20, 30, 50, and 100 mg/mL), sterile water and saline were scanned in a phantom on a rapid switching single-source and dual-source DECT scanners from two different vendors. The phantom was equipped with polyurethane rings simulating three body sizes. The datasets were reconstructed in virtual monoenergetic energy levels (70, 80, 90, 100, 110, 120, 130, and 140 keV), virtual non-contrast images and iodine maps. HU and iodine concentrations were measured by placing ROIs in the iodine solutions.

**Results:**

The iodine concentrations were reproduced with a high degree of accuracy for the single-source DECT (1.8–9.0%), showing a slight dependence on phantom size. The dual source DECT technique showed deviant values (error -33.8 to 12.0%) for high concentrations. In relation to the virtual non-contrast measurements, the images from both vendors were affected by the iodine concentration and phantom size (-127.8 to 539.1 HU). Phantom size did not affect the calculated monoenergetic attenuation values, but the attenuation values varied between the scanners.

**Conclusions:**

Quantitative measurements of post-processed images are dependent on the concentration of iodine, the phantom size and different technologies. However, our study indicates that the iodine maps are reliable for quantification of iodine.

## Key points

- Virtual non-contrast images were affected by phantom size.
- Virtual non-contrast images were affected by high iodine concentrations.
- Virtual monoenergetic images were not affected by phantom size.
- The iodine maps quantified accurate values within clinical iodine concentrations.

## Background

Differentiation of tissue is challenging in conventional computed tomography (CT), as HU values are dependent on photon energy, mass density and tissue attenuation coefficient. For soft tissue in particular, the differences in mass density are subtle. The latter is not unique to one specific type of tissue but related to the atomic composition of the tissue. CT scanning with spectral energy, with simultaneous acquisition of datasets using two x-ray spectra of diverse energies, allows optimisation of contrast in vascular imaging, but also virtual subtraction of bone structures, calcified areas or iodine in contrast-enhanced images [1–4]. Manufacturers provide diverse technologies for acquiring spectral CT data that can potentially give different results. Siemens Healthineers uses a dual source CT while General Electric Healthcare uses one x-ray tube with rapid voltage switching [5].

Siemens Healthineers’ dual source CT system uses two x-ray tubes with diverse voltages placed nearly 90° from one another, with opposing detectors. One advantage of this system is that the voltage, current and filter can be chosen independently for the tube. A disadvantage is that scatter radiation hits the non-corresponding detector. However, the detector elements are able to measure and correct this scatter. To fit the gantry, one of the detectors is smaller than the other, entailing a limited field of view [5].

General Electric Healthcare uses one x-ray tube that rapidly switches between a high and a low tube voltage [5]. The detector collects data for both energy spectra in the projections, and because the interval between the high and low energy detection is small, it provides close to simultaneous data acquisition. The reduced photon output at low voltage, caused by limited cathode electrons, is solved by increasing the sampling interval for low energy data. A disadvantage of using one switching tube is the spectral filtration of the two energy spectra [5].

Dual-energy post-processing techniques include using material-specific methods. Using two diverse x-ray energies in imaging, the linear attenuation coefficient of each substance in the image can discriminate specific materials such as iodine. The iodine content in an image can be measured by using different mathematical algorithms, a three-material decomposition approach for dual source dual-energy CT (DECT) platforms or a two-material decomposition for a single-source DECT platform [4]. For abdominal imaging, the three materials are most commonly soft tissue, fat and iodine, using a three-material decomposition. The process of two-material decomposition uses two materials with different attenuation characteristics, for instance iodine and water [4]. Mathematical algorithms create virtual monoenergetic datasets from dual-energy acquisitions. For iodine, the k edge is 33 keV, resulting in a high attenuation of low-energy photons. Monoenergetic images of 40–50 keV will therefore enhance the contrast between iodine and soft tissue. This may result in improved lesion detection, especially for hypervascular liver lesions or pulmonary emboli [6, 7].

Another advantage of increasing the iodine contrast enhancement is that less iodine contrast media needs to be administrated to the patient. Monoenergetic images of high energies will reduce the visualisation of iodine [8]. High-energy monoenergetic images will also suppress scatter and beam hardening caused by bone and metal [4]. Iodine maps are the coloured areas in the iodine enhancement images. The clinical applications of this method are the detection of perfusion problems in the lung after pulmonary embolism or detection of areas of ischaemia in the myocardium [5]. Quantitative analyses of contrast and non-contrast CT images are used to diagnose diseases, such as renal cell carcinoma. Most of the incidentally detected renal masses are benign cysts that do not require treatment. To distinguish between benign and malignant incidents, one of the elements that sets out recommendations for treatment is ROI measurements in CT images, which is used to evaluate attenuation, enhancement, heterogeneity and homogeneity. When evaluating contrast-enhanced and non-contrast-enhanced CT images of homogeneous renal masses, the benign renal masses will have CT values of between -10 and 20 HU and require no further evaluation [9]. CT imaging is also used to differentiate between benign and malignant adrenal adenoma [10]. For unenhanced CT images, the threshold value for a benign adrenal adenoma is ≤ 10 HU. Contrast-enhanced CT images are used to measure the rate of contrast washout, or loss of contrast, after the injection of contrast. The rate is quantified by measuring the lesion attenuation before injection, 60 s after injection and 10 or 15 min after injection. The washout rate is assumed to be longer for a malignant adrenal lesion than a benign adrenal lesion [10]. DECT offers several ways of improving the imaging with respect to tissue quantification and characterisation, and better visualisation of the iodine contrast. However, differences in the functionality and clinical outcome of the technologies across vendors have not been fully assessed, and few systematic evaluations of the different technologies currently available on the market exist.

The aim of this study was to quantitatively benchmark iodine imaging across specific monoenergetic energy levels, iodine maps and virtual non-contrast images with different phantom sizes and iodine concentrations, and from two vendors, in order to fully investigate accuracy and potential differences in functionality and outcome between the technologies.

## Methods

In this study, five different solutions of iodine contrast agent, sterile water and isotonic sodium chloride dilution were used to evaluate spectral imaging. The solutions of iodine contrast agent, Omnipaque 350 mg I/mL, and sterile water were manufactured in a pharmacy laboratory using a calibrated weight. The contrast agent had a mass density of 1.4 g/mL. Solutions were made with the concentrations 10 mg/mL, 20 mg/mL, 30 mg/mL, 50 mg/mL, and 100 mg/mL. Iodine concentrations < 20 mg/mL are more common in clinical practice, but higher concentrations are also employed, especially in coronary angiography. Very high concentrations of iodine were used to investigate whether there was an upper limit of iodine content that the post-processing methods could handle.

### The phantom

The phantom used was Catphan 605 (Phantom Laboratory, Salem, NY, USA), module CTP682, which is equipped with a small, removable container. To simulate three patient sizes, the phantom was scanned with and without two specific sizes of polyurethane rings (intermediate phantom, anteroposterior diameter 26.2 cm, mediolateral diameter 30.5 cm, circumference 126 cm; and large phantom, anteroposterior diameter 34.1 cm, mediolateral diameter 38.5 cm, circumference 161 cm).

### Scanners and technical parameters

The phantom was positioned in the isocentre of the gantry and scanned on a General Electric Revolution CT scanner (single source spectral imaging) (General Electric Healthcare, Waukesha, WI, USA) and a Siemens Somatom Drive CT scanner (dual source spectral imaging) (Siemens Healthineers, Erlangen, Germany).

A fixed tube current was selected for all scans in order to produce a volumetric CT dose index (CTDI_vol_) of 15 mGy. The kernel and iterative reconstruction algorithms used for each scanner were the same as those normally used for abdominal CT scanning in hospitals: standard low-frequency kernel in combination with Adaptive Statistical Iterative Reconstruction-V 50% for the General Electric Revolution scanner and I30F kernel in combination with Advanced Modelled Iterative Reconstruction 3 for the Siemens Drive scanner. Furthermore, the abdominal protocol for Siemens Drive includes tin filtration to improve the dual-energy spectral separation. The scan parameter settings are presented in Table 1.

**Table 1:** Scan parameter settings used for phantom scans for all phantom sizes and iodine concentrations

	Tube voltage (kV)	Rotation time (s)	Tube current (mA)	Pitch	Collimation (mm)	Slice width (mm)	Kernel	(DFOV) (mm)	CTDI_**vol**_ (mGy)
General Electric Revolution	80/140	0.5	320	0.516	40	2.5 plus	Standard	400	15
Siemens Somatom Drive	80/Sn140	0.5	400/155	0.5	12	3	I30f	210	15.1

*DFOV* Display field of view, *CTDI*_*vol*_ Volumetric computed tomography dose index, *GE* General Electric, *Sn* Tin filter

### Post-processing

All images were post-processed, viewed and analysed on the proprietary workstation: AW server (General Electric Healthcare, WI, USA) was used to post-process the General Electric images, while Syngo.via (Siemens Healthineers, Erlangen, Germany) was used to post-process the Siemens images.

The datasets were reconstructed in virtual monoenergetic energy levels representing the following photon energy levels: 70, 80, 90, 100, 110, 120, 130, and 140 keV. Iodine maps and virtual non-contrast enhanced images, VUE (General Electric) and VNC (Siemens), were reconstructed. In virtual unenhanced reconstructions, the iodine is subtracted from the voxels. However, General Electric adds the value of blood as a substitute for the iodine values [11].

### Image analysis

**Fig. 1:**
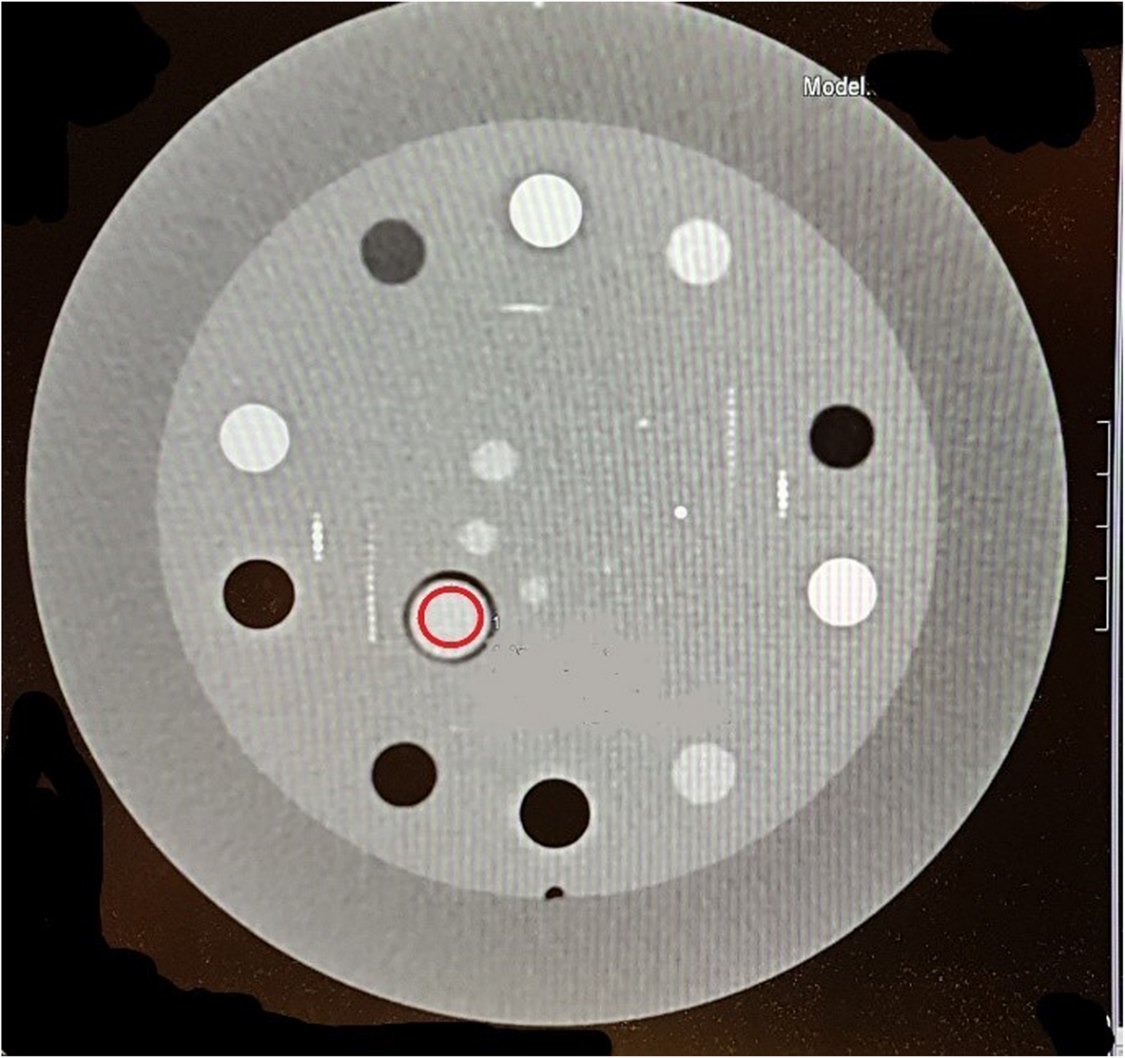
The Catphan CTP682 phantom was positioned in the isocentre of the gantry. The regions of interest were placed in the centre of the iodine solution in the phantom, here visualised as a red ring in the image

The attenuation level (HU) and iodine concentration were measured by placing regions of interest (ROI) within the specific solutions, as shown in Fig. 1. The mean HU values were recorded by placing circular ROIs in the middle of the removable container of the specific solutions. All ROI measurements were performed three times in the same image. The ROIs were defined manually, and the sizes were kept between 0.8 and 0.9 cm^2^ for all measurements.

### Statistical analysis

The programme used for the statistical analyses was Stata 16. The analyses were performed using quadratic regression models for each solution of iodine contrast with linear and quadratic terms of energy and machine or phantom size as fixed effects. Marginal means were estimated to test the difference between phantom sizes or machines and reported estimates with a 95% confidence interval. If no other ways were defined and used for all comparisons, *p* ≤ 0.05 was indicative of a statistically significant difference.

## Results

### Iodine concentration calculations

Table 2 shows the measured concentrations of iodine for the different dilutions of iodine, sterile water and sterile saline, for all phantom sizes. The measured iodine concentration in water and saline were close to 0. The measured values in the iodine dilutions were close to the nominal concentrations, except for the highest concentration calculated by Siemens. The error varied from 1.8 to 9.0% for General Electric, and -33.8 to 12.0% for Siemens.

**Table 2:** Iodine concentration measurements for all concentrations (mg/mL) and phantom sizes

	General Electric Revolution			Siemens Somatom Drive		
Solution	Small	Medium	Large	Small	Medium	Large
Water	-0.2	-0.1	-0.9	0.1	0.2	0.3
Saline	-0.2	-0.6	-0.7	0.4	0.2	0.3
Iodine 10 mg/mL	10.3 (3.0)	10.7 (7.0)	10.9 (9.0)	11.2 (12.0)	11.1 (11.0)	11.2 (12.0)
Iodine 20 mg/mL	20.6 (3.0)	21.0 (5.0)	21.7 (8.5)	21.8 (9.0)	21.6 (8.0)	22.2 (11.0)
Iodine 30 mg/mL	30.8 (2.7)	31.5 (5.0)	32.4 (8.0)	31.7 (5.7)	31.8 (6.0)	32.2 (7.3)
Iodine 50 mg/mL	51.4 (2.8)	52.8 (5.0)	52.2 (4.4)	51.0 (2.0)	51.0 (2.0)	52.0 (4.0)
Iodine 100 mg/mL	101.8 (1.8)	104.0 (4.0)	103.1 (3.1)	66.2 (-33.8)	73.2 (-26.8)	76.6 (-23.4)

Percentage difference between known and measured iodine concentration in parentheses

*CTDI*_*vol*_ Volumetric computed tomography dose index

### Virtual non-contrast images

Table 3 shows the HU measurements from the virtual non-contrast-enhanced images. The attenuation values ranged between -127.8 and 7.1 for General Electric, and -7.2 and 539.1 for Siemens. With increasing iodine concentration, General Electric shifted the attenuation values towards negative values, while Siemens shifted towards more positive values. The HU values for both systems had greater divergence for the higher concentrations of iodine. With the General Electric system and with iodine concentrations of 20 mg/mL or more, the HU changed with phantom size. The corresponding HU values for Siemens were less affected by the phantom size, except for the highest concentration of iodine.

**Table 3:** HU and standard deviation (SD) in virtual non-contrast enhanced images for all concentrations and different phantom sizes

	General Electric Revolution			Siemens Somatom Drive		
Solution	Small	Medium	Large	Small	Medium	Large
Water	-1.5±5.0	-2.7±13.8	-8.4±16.0	-3.7±3.0	-6.4±5.6	-4.6±8.5
Saline	7.1±5.8	2.4±16.2	1.3±16.9	1.9±3.1	2.4±7.2	1.5±8.0
Iodine 10 mg/mL	5.4±3.0	-6.7±5.8	-4.3±9.6	-7.2±2.7	-7.1±5.8	-5.2±8.3
Iodine 20 mg/mL	-1.3±3.1	-24.5±15.2	-30.3±10.4	-2.3±4.4	-0.8±6.1	-3.7±9.8
Iodine 30 mg/mL	-13.1±3.1	-30.3±5.9	-48.2±9.5	9.5±4.8	8.4±9.6	3.1±9.5
Iodine 50 mg/mL	-26.9±4.2	-63.5±8.8	-75.4±11.7	39.7±11.6	35.0±12.0	21.5±16.6
Iodine 100 mg/mL	-64.6±4.3	-121.6±8.5	-127.8±14.5	539.1±39.2	420.1±34.6	367.4±22.4

*HU* Hounsfield units

### Virtual monoenergetic images

**Fig. 2:**
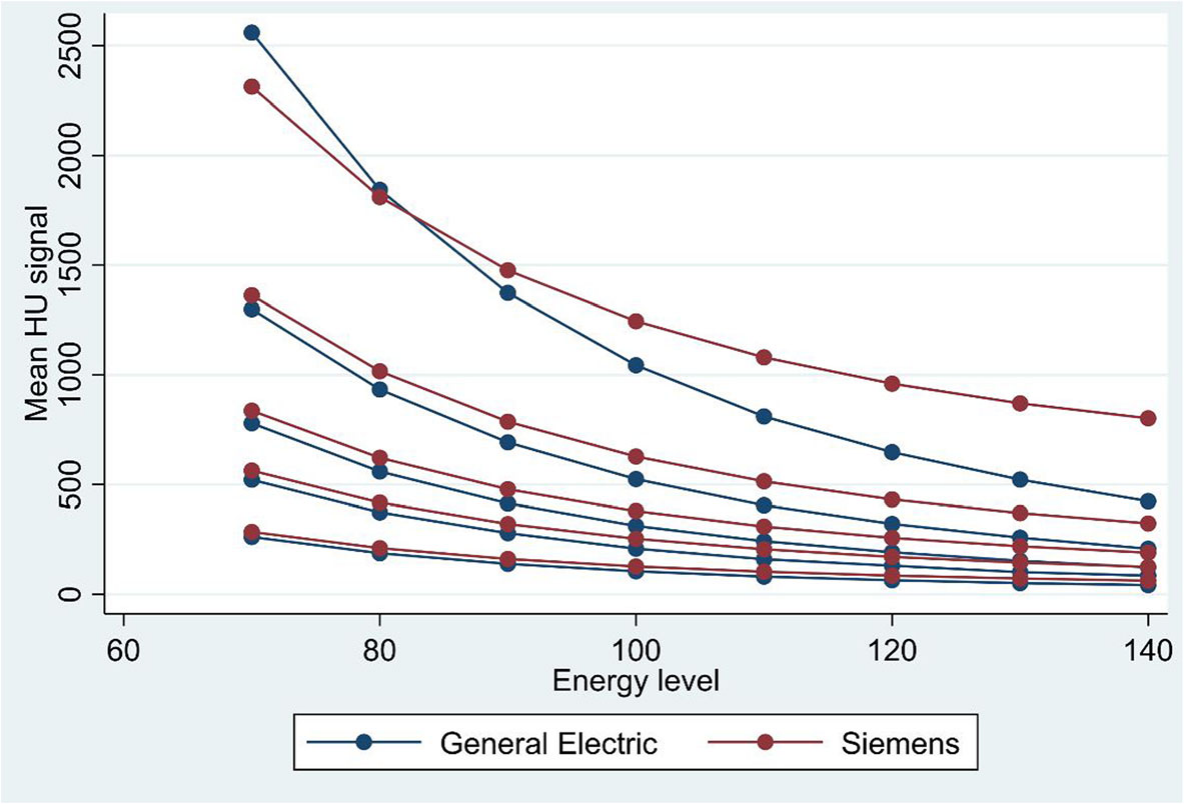
The mean HU in reconstructed monoenergetic images for each concentration of iodine. The virtual monoenergetic energy levels have the unit keV. *HU* Hounsfield units

Tables 4 and 5 show the HU in the solutions in the reconstructed monoenergetic images from General Electric and Siemens. Tables 6 and 7 show the mean difference in HU between phantom sizes for all monoenergetic images for each iodine concentration, for both vendors. There was no significant difference (*p =* 0.158*–p* = 0.999) in measured attenuation values between the phantom sizes within each iodine concentration, except for the lowest concentration of iodine using General Electric technology (*p* = 0.036). Table 8 shows the mean difference in HU between the vendors for all monoenergetic images with each iodine concentration. For iodine concentrations of between 10 and 50 mg/mL, the calculated HU from monoenergetic images from Siemens Drive was significantly different than calculated HU from monoenergetic images from General Electric Revolution (*p* < 0.001). This is visualised in Fig. 2. For the iodine concentrations of 100 mg/mL, there was a shift in the calculations of HU, where HU in monoenergetic images from General Electric showed stronger energy dependence with HU at 70 keV and 80 keV, measuring higher HU than for Siemens (*p* ≤ 0.032). For higher energy levels, Siemens calculated the highest HU.

**Table 4:** Measured HU values in monoenergetic images with General Electric Revolution CT

Phantom size	Energy level (kV)	Water	Saline	10 mg/mL	20 mg/mL	30 mg/mL	50 mg/mL	100 mg/mL
Small	70	-1.6	6.8	261.1	520.6	777.9	1292.6	2556.5
	80	0.1	8.5	189.3	375.8	562.2	937.4	1850.8
	90	1.1	9.4	142.0	281.2	420.9	702.2	1388.6
	100	1.7	10.1	109.1	215.7	322.4	538.0	1065.1
	110	2.3	10.4	85.7	169.5	254.2	422.6	834.4
	120	2.7	10.7	69.5	137.1	205.4	341.7	673.9
	130	2.9	11.2	56.6	111.6	166.2	278.9	551.9
	140	3.0	11.3	49.0	92.2	138.4	229.2	457.8
Medium	70	-2.6	3.3	258.0	520.7	775.2	1300.5	2565.4
	80	1.7	7.7	183.4	370.6	555.9	930.6	1843.2
	90	5.3	10.6	134.8	280.3	411.7	689.4	1370.0
	100	8.0	12.3	101.6	203.3	310.6	522.1	1038.4
	110	9.6	14.2	77.0	154.4	240.5	401.6	802.4
	120	10.9	16.5	60.7	132.5	189.5	316.6	638.3
	130	12.5	15.5	47.8	95.9	151.8	254.4	511.6
	140	13.2	17.4	37.6	87.7	121.2	203.4	413.4
Large	70	-7.6	1.8	266.8	524.4	785.4	1305.1	2556.4
	80	-2.1	7.1	190.4	375.3	559.9	932.6	1836.1
	90	2.2	11.2	141.0	276.3	410.7	685.0	1360.5
	100	6.6	12.7	105.2	206.8	303.3	513.6	1029.1
	110	8.6	13.4	80.4	158.1	233.0	392.8	794.6
	120	9.6	14.8	63.2	123.1	181.8	305.7	630.9
	130	11.0	15.3	49.5	96.8	141.7	242.6	504.0
	140	12.2	16.1	40.4	76.3	110.8	192.0	401.3

*HU* Hounsfield units

**Table 5:** Measured HU values in monoenergetic images with Siemens Somatom Drive

Phantom size	Energy level (kV)	Water	Saline	10 mg/mL	20 mg/mL	30 mg/mL	50 mg/mL	100 mg/mL
Small	70	-0.1	11.4	286.7	566.3	835.9	1362.2	2263.3
	80	0.6	10.1	212	419.9	621.4	1017.3	1795.6
	90	1.0	9.3	162.4	322.6	478.9	788.2	1483.9
	100	1.3	8.7	128.1	255.3	380.5	629.4	1268.4
	110	1.5	8.3	103.7	207.5	310.4	516.7	1115.0
	120	1.7	8.0	85.9	172.6	259.3	434.4	1003.0
	130	1.8	7.7	72.6	146.5	221.1	373	919.5
	140	1.9	7.6	62.5	126.7	192	326.2	856.0
Medium	70	-2.5	8.0	280.6	559.2	832.1	1357.2	2324.5
	80	-1.9	7.7	207.5	414.9	618.6	1013	1812.7
	90	-1.6	7.6	158.8	318.7	476.3	783.5	1471.5
	100	-1.3	7.5	125.0	252.4	378.0	624.9	1236.1
	110	-1.1	7.4	101.1	205.0	308.0	512.3	1068.2
	120	-1.0	7.3	83.6	170.5	257.0	430.0	945.8
	130	-0.9	7.3	70.6	144.7	218.9	368.7	854.5
	140	-0.8	7.3	60.7	125.1	189.9	321.9	785.0
Large	70	0.6	8.8	287.8	565.2	841.7	1367.6	2350.1
	80	0.9	8.2	213.4	418.8	625.0	1020.2	1823.5
	90	1.2	7.6	163.8	321.1	480.5	788.5	1472.5
	100	1.3	7.2	129.4	253.5	380.2	628.6	1230.3
	110	1.4	6.9	105.2	205.4	308.9	514.8	1057.8
	120	1.5	6.6	87.5	170.4	256.8	431.8	931.3
	130	1.6	6.5	74.3	144.3	218.0	369.8	836.8
	140	1.6	6.3	64.2	124.4	188.5	322.6	765.1

*HU* Hounsfield units

**Table 6:** Mean difference in HU values between phantom sizes for all monoenergetic images and iodine concentrations with General Electric Revolution CT

Phantom sizes	10 mg/mL	20 mg/mL	30 mg/mL	50 mg/mL	100 mg/mL
M vs S	-7.68 (0.036)	-7.30 (0.294)	-11.42 (0.281)	-15.51 (0.368)	-24.54 (0.457)
L vs S	-3.17 (0.365)	-8.33 (0.233)	-15.13 (0.158)	-21.65 (0.213)	-33.26 (0.316)
L vs M	4.52 (0.201)	-1.030 (0.880)	-3.71 (0.719)	-6.14 (0.719)	-8.72 (0.790)

*HU* Hounsfield units, *M vs S* Medium *versus* small phantom size, *L vs M* Large *versus* medium phantom size, *L vs S* Large *versus* small phantom size

*p*-value in parentheses

**Table 7:** Mean difference in HU values between phantom sizes for all monoenergetic images and iodine concentrations with Siemens Somatom Drive

Phantom sizes	10 mg/mL	20 mg/mL	30 mg/mL	50 mg/mL	100 mg/mL
M vs S	-3.25 (0.334)	-3.36 (0.607)	-2.59 (0.788)	-4.49 (0.772)	-25.80 (0.333)
L vs S	1.46 (0.661)	-1.79 (0.784)	0.01 (0.999)	-0.44 (0.997)	-29.66 (0.268)
L vs M	4.71 (0.167)	1.58 (0.809)	2.6 (0.787)	4.05 (0.793)	-3.86 (0.883)

*HU* Hounsfield units, *M vs S* medium *versus* small phantom size, *L vs M* large *versus* medium phantom size, *L vs S* large *versus* small phantom size

*p*-value in parentheses

**Table 8:** Mean difference in HU values between the vendors for all monoenergetic images and iodine concentrations

	10 mg/mL	20 mg/mL	30 mg/mL	50 mg/mL	100 mg/mL
Mean HU difference	21.98 (< 0.001)	42.69 (< 0.001)	64.47 (< 0.001)	98.85 (< 0.001)	-222.65–358.41 (0.032)

*HU* Hounsfield units

*p*-value in parentheses

## Discussion

A benefit from DECT is improved tissue quantification [1–5]. One example in the clinical setting is the use of quantification in characterisation of renal incidents to decide malignancy [9]. A prerequisite is that diverse technologies calculate equal values. This is crucial to be able to make the right diagnosis if a patient during a follow-up is examined with different CT technology. Still, different technologies between vendors might result in diverse values. The following will discuss the DECT quantitative methods using virtual monoenergetic images, virtual non-contrast images and iodine maps, performed with DECT technologies from General Electric and Siemens.

### Iodine concentration calculations

Several clinical studies have measured iodine concentrations in the blood to be in the range of 1–30 mg/mL [12–14]. Our study included iodine concentrations from 0 to 100 mg/mL to fully investigate and benchmark the calculated concentration within the clinical range, but also higher concentrations to investigate potential limitations for the specific techniques.

The technique from General Electric calculated the iodine concentrations with a high degree of accuracy, with a deviation of 1.8 to 9.0% from nominal values. The calculated values of the iodine concentration of 10 mg/mL showed a deviation of 3% and 9% for the small and large phantom respectively, indicating a slight dependence on phantom size for the lowest iodine concentrations. However, the percentage deviations at the lowest concentrations should be read carefully, as small changes in low concentration dilutions will lead to high percentage deviations, which is also supported by other studies [12].

The technique from Siemens calculated the iodine concentrations with a deviation of 2.0–12.0% from nominal value in the range of 10–50 mg/mL iodine. For the highest iodine concentration level, 100 mg/mL, Siemens showed a large deviation (-33.8%), indicating a limit of the system for the highest concentrations. However, this is outside the concentration range normally used for clinical purposes. A possible explanation is that the signal in the voxels is saturated. Another explanation could be that the post-processing method fails in the spectral separation, especially in the low-energy datasets. This is also visible in the quantified HU values in the reconstructed monoenergetic images with low-energy levels. A study by Pelgrim et al. [13] investigated the accuracy of iodine quantification in the concentration range 0–50 mg/mL using two DECT systems. The study showed a high degree of accuracy between measured and true concentration and concluded that it was a good method for quantifying perfusion in the myocardium. However, it also concluded that caution should be shown when comparing the quantification between different scanning techniques.

Our study indicates that iodine maps are a potential quantitative method that can be used in diagnostics. This is supported by numerous studies, suggesting that iodine concentration quantification may be a potential way of improving the evaluation of differences in iodine uptake and blood supply between benign and malignant lesions [14–18]. Stiller et al. [14] concluded in their study that quantitative iodine maps could potentially replace perfusion imaging.

### Virtual non-contrast images

Several studies show varying conclusions whether virtual non-contrast images can eliminate the need for the pre-contrast, unenhanced CT scan series. Bae et al. [19] quantitatively measured that the VNC image derived from dual source DECT was comparable to the TNC image in effectiveness to detect biliary stones in 45 patients and considered VNC as acceptable replacement for the TNC image. Li et al. [20] investigated abdominal tissue and vascular attenuation values on VUE and TNC images of patients with BMI < 25. The images were performed with a single-source DECT, and the image sets were not significantly different. Jamali et al. [21] performed a quantitative comparison of true unenhanced and virtual unenhanced abdominal images using a dual layer detector. The two data sets demonstrated statistically significant differences in attenuation values in the spleen, muscle, kidney and fat. A study of Lehti et al. [22] investigated the aortic attenuation in 30 patients to assess whether VNC images could replace TNC images. All images were performed using a dual source DECT. All VNC images showed a significant higher attenuation than the TNC images, attributed to incomplete iodine suppression. The VNC images did also remove structures like calcification in the aortic wall and part of stents. Borhani et al. [23] reviewed abdominal images performed with single-source DECT from 94 patients. An intra-patient analysis showed that the attenuation values between VUE and TNC images were significantly different in several measured tissues and were severely different in the aorta and the right adrenal gland.

Eliminating the signal from iodine in the image should leave the HU at the organ value, in our study water or blood. The technique from General Electric replaces the iodine signal with blood, which is about 30 HU [24]. The technique from General Electric showed a virtual non-contrast attenuation value range of between -127.8 and 7.1 HU. None of the virtual non-contrast calculations were close to the HU of blood, and for higher concentrations, the attenuation values diverged greatly, indicating a limit to the system’s ability to eliminate the signal from high concentration materials. There were also indications that phantom size influenced the ability to eliminate the signal of iodine.

The HU calculated from the Siemens technique showed small variations at around HU = 0 in the range 10–30 mg/mL iodine (-0.8 to 9.5 HU), but no dependence on phantom size. For the concentration 100 mg/mL iodine, the attenuation values diverged greatly from zero, thus also indicating a limit to the system’s ability to eliminate the signal from high-concentration materials.

For the dilution 100 mg/mL iodine, none of the post-processing methods gave a precise result, possibly due to beam hardening. Other studies [22, 23] have similar findings in the arterial phase with high iodine concentrations. The results from this study, shown in Table 3, indicate that HU values in virtual non-contrast images are not as reliable as measurements of iodine concentration. Our results indicate that iodine maps may be more suitable for malignancy evaluation, whereas virtual non-contrast images may be more applicable in qualitative assessments. Threshold values for iodine concentration measurements in malignancy evaluation are therefore needed.

### Virtual monoenergetic images

Marin et al. [4] suggest several clinical applications of virtual monoenergetic images, including characterising renal cysts and detecting hypervascular liver lesions. The virtual monoenergetic images can generate lesion-specific spectral attenuation curves based on the attenuation across a large spectrum of synthesised monoenergetic energies. Several studies concluded that these curves may assist in the characterisation of renal and liver lesions [25, 26]. A precondition, however, is that the virtual attenuation values are consistent. Our study shows that the attenuation values for the iodine solutions were not significantly different between the phantom sizes, meaning that the monoenergetic images provide consistent iodine HU values regardless of the phantom size. One exception was the calculated HU value of the iodine 10 mg/mL solution from General Electric Revolution. There was a significant difference between HU values using a small phantom size *versus* medium phantom size, but there was no difference in HU value between small and large, or medium and large phantom sizes. The significant inequality may be coincidental. For the water and saline, there were differences in the measured HU value between the phantom sizes. This may be due to an increase in noise and photon starvation with increasing phantom size, since the same CTDIvol level was used independently of phantom size, in addition to beam hardening effects with increasing phantom size. The increase in noise for low monoenergetic energy levels are well documented in other studies [25, 27, 28]. Similar studies need to be performed with iodine solutions of several concentrations in the range 1–10 mg/mL, and even larger phantoms.

The vendors use distinctive methods to calculate the HU values from virtual monoenergetic images. The spectral separation can be diverse using a single source than a dual source x-ray tube. Our study shows that the calculated HU values from monoenergetic images from Siemens Somatom Drive are significantly different from the HU values calculated from monoenergetic images from General Electric Revolution. It is important that users are aware of this distinction if the purpose is to use absolute values. The shapes of the curves are similar, but as the iodine concentration increases, the divergence in the HU between the vendors rises. The iodine concentration of 100 mg/mL is out of the clinical range, yet it is interesting that the HU values in the monoenergetic energy levels vary to a significant extent between the two vendors.

### Study limitations

This was a phantom study, without anatomical noise or movement artefacts from breathing and pulsation. To fully assess the potential benefits of simultaneous dual-energy uptake, the results from this study should be leveraged in a clinical setting. It is important to emphasise that this study investigated the quantification accuracy of post-processing methods, and not the qualitative opportunities. The iodine solutions used in this study were of high concentration to test the limits of the systems, and it would be interesting to use the same method to evaluate virtual non-contrast and monoenergetic images with lower iodine concentrations of 1–10 mg/mL. Other DECT techniques on the market could give different results to those obtained from this study. Evaluation of those techniques was not part of this study. Another consideration worth mentioning is the display field of view (DFOV). For the General Electric scanner, a 40-cm DFOV was used for measurements, while a 21-cm DFOV was used for Siemens. However, this will affect the spatial resolution rather than the attenuation, which was the focus of this study.

## Conclusions

Our study showed that quantitative measurements of post-processed images are affected by both iodine concentration and phantom size. The attenuation values from virtual monoenergetic images were significantly different between the two technologies. Nonetheless, the measured iodine concentrations were close to the nominal concentrations within clinical range for both vendors, so our study indicates that the iodine maps are more suited to the quantification of iodine than HU measurements. To fully assess the potential benefits of simultaneous dual-energy uptake, the results from this study should be leveraged in a clinical setting.

## Data Availability

The datasets analysed in the present study are available from the corresponding author on reasonable request.

## Abbreviations

CT: Computed tomography
CTDI_vol_: Volumetric CT dose index
DECT: Dual-energy CT
DFOV: Display field of view
ROI: Region of interest
TNC: True non-contrast
VNC: Virtual non-contrast
VUE: Virtual unenhanced
